# Structural and Chemical Analysis of Three Regions of Bamboo (*Phyllostachys Edulis*)

**DOI:** 10.3390/ma17205027

**Published:** 2024-10-14

**Authors:** Shaohua Gu, Ana Lourenço, Xin Wei, Jorge Gominho, Ge Wang, Haitao Cheng

**Affiliations:** 1 International Center for Bamboo and Rattan, Beijing 100102, China; gushaohua@icbr.ac.cn (S.G.); wangge@icbr.ac.cn (G.W.); 2 Centro de Estudos Florestais & Laboratório TERRA, Instituto Superior de Agronomia, Universidade de Lisboa, 1349-017 Lisboa, Portugal; 3 College of Furniture and Art Design of Furniture and Art Design, Central South University of Forestry and Technology, Changsha 410004, China; t20222655@csuft.edu.cn

**Keywords:** bamboo, gradient structure, physicochemical properties, biorefinery, *Phyllostachys edulis*

## Abstract

This study focuses on three different regions of moso bamboo (Phyllostachys edulis): an inner layer (IB), middle layer (MB), and outer layer (OB), to comprehensively characterize the structural features, chemical composition (ash, extractives and lignin contents), and the lignin monomeric composition as determined by analytical pyrolysis. The results show that bamboo presents a gradient structure. From the IB to OB, the vascular bundle density and fiber sheath ratio increase, the porosity decreases (from 45.92% to 18.14%), and the vascular bundle diameter–chord ratio increases (from 0.85 to 1.48). In terms of chemical composition, the ash, extractives, and acid-soluble lignin content gradually decrease from IB to OB. The holocellulose content follows the trend: MB (66.3%) > OB (65.9%) > IB (62.8%), while the acid-insoluble lignin content exhibits the opposite trend: IB (22.6%) > OB (17.8%) > MB (17.7%). Pyrolysis products reveal the diversity of carbohydrates and lignin derivatives, with a lignin monomeric composition rich in syringyl and guaiacyl units and lower amounts of H-units: the IB has an H:G:S relation of 18:26:55, while 15:27:58 is the ratio for the MB and 15:40:45 for the OB; S/G ratio values were, respectively, 1.22, 1.46, and 0.99. A comprehensive analysis highlights significant gradient variations in the structure and chemistry of bamboo, providing robust support for the classification and refinement methods of bamboo residues for potential applications.

## 1. Introduction

*Phyllostachys edulis*, also known *Phyllostachys pubescens* and *Phyllostachys heterocycle*, commonly referred to as moso bamboo, is a member of the *Bambusoideae* subfamily within the *Poaceae* family. Its widespread recognition underscores its global importance as a key bamboo species used for several industries in China and other countries, for example, in traditional medicine, the textile industry (production of rayon), furniture, construction, pulp and paper, musical instruments, and food additives [1].

**Figure 1:**
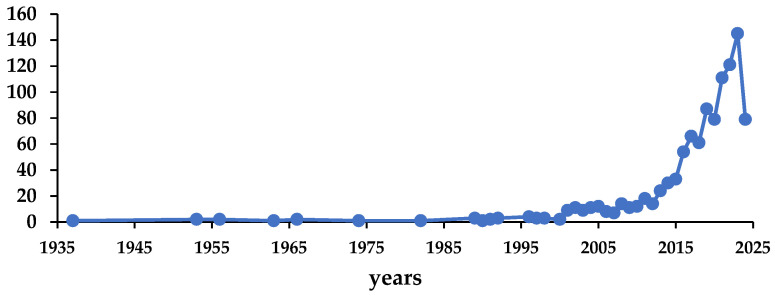
Number of publications about bamboo across the years. Source: Scopus; keyword: *Phyllostachys edulis*.

**Figure 2:**
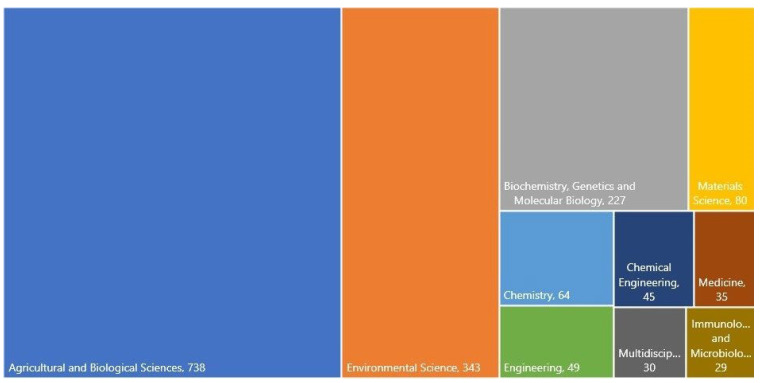
Tree map including the main subject areas of publication. Source: Scopus (July 2024); keyword: *Phyllostachys edulis*. Legend: Multidiscip—Multidisciplinary; Immuno and Microbiolo—Immunology and Microbiology.

To have an idea about the studies on bamboo, a research was made in Scopus data base using *Phyllostachys edulis* as a keyword. The results are presented in Figure 1 and Figure 2. It is clear that there is an increasing interest in bamboo, as shown in Figure 1, where the publications have increased from 1 article in 1937 to about 44 articles published this until July 2024. The subject area, according to the Scopus definition, are diverse; the main ones are as follows: agricultural and biological sciences (number of publications: 738), environmental sciences (343), biochemistry, genetics, and molecular biology (227), material science (80), chemistry (64), engineering (49), chemical engineering (45), medicine (35), multidisciplinary (30), and immunology and microbiology (29), as can be seen in Figure 2. Notably, the global bamboo industry makes a significant economic contribution. For example, in China, the trade volume of bamboo products reached $2.645 billion in 2021. Scholars predict that, with policy support, the total output value of the bamboo industry will exceed $137 billion by 2035. This economic context further highlights the practical importance of bamboo research and its relevance to sustainable development [2].

In total, only 12 reviews were published; for example, the use of bamboo for medium-density fiberboards panels was evaluated [3], as well as for pulp production [4] or even the use of leaves for the extraction of compounds with interest [5]. In fact, across the years, several studies have focused on more valorization routes for this species and different parts of the plant, in particular, with respect to its chemical components, such as extractives (flavonoides) and their antioxidant activity [6] and, more recently, its application in fiber-reinforced composites [7] or synthetic fibers [1].

The wide applications of bamboo are attributed to its anatomical and chemical characteristics. From an anatomical perspective, the bamboo culm is characterized by the presence of vascular bundles and parenchyma tissue [8]. Vascular bundles provide strength to bamboo and consist of one or two protoxylems, two large metaxylem vessels, and a thin-walled phloem composed of sieve tubes linked to companion cells [9]. Surrounding these vital elements are sclerenchymatous fibers, providing structural support. The arrangement and composition of these components differ both radially and axially within the culm, resulting in variations in bamboo properties across species and in different directions [10,11]. Recently, a concentration of vascular bundles in the epidermal side, producing mechanical strength more effectively compared to an uniform distribution, was demonstrated [12].

Therefore, bamboo species presents different characteristics from the inner to outer cross-section, displaying a transitional and non-uniform structure where one form of structure, component, or phase gradually transitions to another. Simultaneously, its microscopic structure, physicochemical properties, and mechanical strength parameters undergo stepwise changes [13,14]. These properties enable their specialized use in various industrial applications. For example, the inner layer, with its larger and more sparse pore structure, facilitates matrix infusion, imparting light-transmitting, magnetic, and fluorescent properties suitable for doors, windows, and architectural decorations [15], and its high cellulose content makes it ideal for paper and pulp production [16]. The middle layer, with moderate density and porosity, offers flexibility and toughness, making it perfect for furniture manufacturing [17], and its higher sugar content and easier removal of hemicellulose and lignin make it advantageous for bioethanol production [18]. The outer layer, with dense vascular bundles and fiber sheaths, provides toughness and creep resistance, making it suitable for producing stable bamboo–wound composite pipes and special-shaped structural components in construction [19,20,21]. Understanding these differences is crucial for optimizing bamboo’s utilization and enhancing its value as a sustainable resource, from durable structural materials to bioenergy and biodegradable products.

From a chemical perspective, moso bamboo possesses a lignin content ranging from 14.6% to 29.1%, comprising three structural units: guaiacyl (G), syringyl (S), and p-hydroxyphenyl (H) [22,23,24]. Lignin is a three-dimensional macromolecule formed by carbon–carbon and ether linkages of benzyl propane-type monomers, containing various active functional groups such as hydroxyl, aldehyde, ketone, carboxyl, and methoxy. These structural characteristics designate it as a potential raw material for high-value-added chemical products, particularly phenolic compounds. Meanwhile, the holocellulose content ranges from 60% to 70%, making it suitable for the production of carboxymethyl cellulose, xylitol, and bamboo charcoal [25,26]. These products find wide applications, including as dispersants, emulsifiers, xylitol sweeteners, and adsorbents, among other uses. Additionally, bamboo contains abundant organic compounds, including polysaccharides and salicylic acid. The pressing method to extract fresh bamboo juice in China can be employed for brewing distinctive bamboo juice wine or health beverages. Therefore, the residual materials from bamboo processing have become a noteworthy resource in biorefinery, serving high-value purposes in the production of chemicals and materials.

Overall, the gradient structure of bamboo material results in significant variations in chemical composition across different sections. This variation may be considered a drawback, especially when aiming to provide stable and consistent raw materials for industrial purposes. However, it can also be regarded as a source of almost limitless diversity in compounds, leading to a broader range of potential bio-based products. Currently, there is limited systematic research on the correlation between the different parts of bamboo, its chemical composition and, in particular, lignin. It is essential to conduct detailed investigations into the chemical components and lignin monomers of these three regions to advance the process of “bamboo biorefinery”. In this study, the structural features and chemical analyses were performed on the following different radial parts of bamboo called here: inner bamboo (IB), middle bamboo (MB), and outer bamboo (OB) to comprehensively understand their characteristics by summative analysis and analytical pyrolysis (Py-GC/MS). This research not only provides theoretical guidance for biomass pyrolysis liquefaction technology but also offers technical support for catalytic pyrolysis aimed at producing high-value-added chemical products.

## 2. Materials and Methods

### 2.1. Sample Preparation

**Figure 3:**
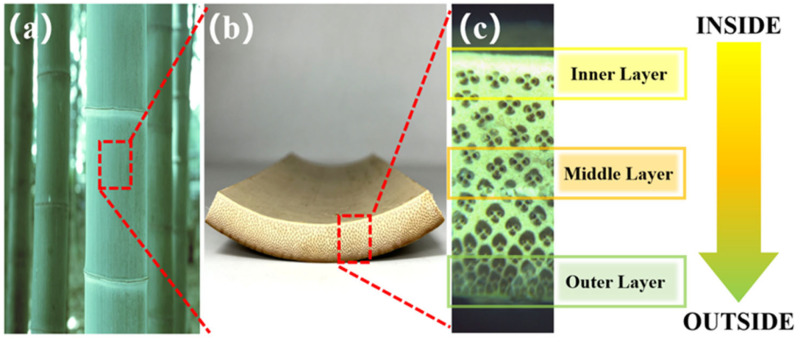
The preparation of bamboo sample: (**a**) bamboo culm, (**b**) bamboo strip, and (**c**) bamboo cross-sectional structure.

The bamboo used in this study was *Phyllostachys edulis*, sourced from Yibin City, Sichuan Province, China. The bamboo was one year old, with a height of 9 m, an average breast diameter of 10 cm, and a thickness of 16 mm. The bamboo stalks were harvested at approximately 1.5 m above the ground from the whole bamboo culm, serving as the experimental material. The bamboo stalks were divided into an average of 5 layers from the inner to the outer layers. Three layers, representing IB, MB, and OB, were selected for experimentation, as presented in Figure 3. The bamboo components underwent knife milling using a Retsch SM2000 grinding machine (Retsch, Haan, Germany) and were sieved and sealed after obtaining bamboo powder with a particle size ranging from 40 to 60 mesh. The samples were then placed in a constant temperature and humidity chamber (25 °C, relative humidity 65%) to equilibrate moisture content.

### 2.2. Microstructural Analysis

The complete bamboo samples were subjected to micro-CT scanning (nano Bruker, Billerica, MA, USA) with a precision of 4.5 μm. The original shadow projections were reconstructed into multiple sets of parallel X-ray micrographs. Individual vascular bundles after gold sputtering were characterized using a scanning electron microscope (Quanta2000, PHILIPS Ins., Amsterdam, The Netherlands) with an accelerating voltage of 7.5 KV.

### 2.3. Summative Chemical Analysis

The chemical composition of the three regions, namely ash, total extractives, total lignin, and holocellulose, were determined following methods cited in the literature [27]. Ash content was measured by weighing the residue after overnight incineration at 525 °C, following the TAPPI standard T211om-93 [28]. The total extractives content was determined through successive extractions using dichloromethane, ethanol, and water in a Soxhlet apparatus. Dichloromethane was used for 6 h, followed by ethanol (16 h) and water (16 h). The extracts from the extraction thimbles were quantified by weighing after each extraction, based on the methods described in the literature [29,30]. The acid-soluble and Klason lignin were determined on extractive-free materials. Klason lignin was determined through acid hydrolysis following the TAPPI standard [31]. To a 0.35 g sample, sulfuric acid (72%, 3.0 mL) was added, stirred for 1 h at 30 °C, diluted to a 3 wt% H_2_SO_4_ concentration, and further hydrolyzed for 1 h at 120 °C. The Klason lignin was determined by weighing the mass of solid residue after filtration in a G4-crucible, thorough washing with distilled water, and subsequent drying in an oven. Klason lignin was quantified as the mass of solid residue corrected for ash content, as described above. The soluble lignin was determined by UV-spectrophotometry, measuring the absorbance of the hydrolysis products of the Klason lignin procedure at 205 nm, following the TAPPI standard [32]. The total lignin content was calculated as the sum of acid soluble and Klason lignin. Hollocellulose was determined by chlorite method as described in Miranda et al. [30].

### 2.4. Pyrolysis-GC/MS

The three bamboo extractives-free samples and their correspondent Klason lignins were ball-ground to powder in a Retsch MM200 mixer ball (Retsch, Haan, Germany). The powder was subsequently dried in a vacuum oven under phosphorus pentoxide conditions. Approximately 0.15 mg of each sample underwent pyrolysis at 550 °C (1 min) using a 5150 CDS apparatus (Oxford, MO, USA) connected to a gas chromatographer (Agilent GC 7980B, Wilmington, USA), which was equipped with a ZB-1701 fused-silica capillary column (60 m × 0.25 mm i.d. × 0.25 µm film thickness) and linked to an Agilent 5977B mass-selective detector (Wilmington, DE, USA) using EI at 70 eV. The GC oven temperature was programmed as follows: started at 40 °C (held for 4 min), increased to 70 °C at a rate of 10 °C/min, further raised to 100 °C at 5 °C/min, then to 265 °C at 3 °C/min (held for 3 min), and finally to 270 °C at 5 °C/min (held for 9 min). The injector and GC/MS interface were maintained at temperatures of 270 and 280 °C, respectively. Helium with a total flow of 1 mL/min was employed as the carrier gas. Compound identification was performed by comparing the mass-to-charge ratio (*m*/*z*) spectra with Wiley, NIST libraries, and the literature [33].

## 3. Results and Discussion

### 3.1. Physical Anatomical Properties

**Figure 4:**
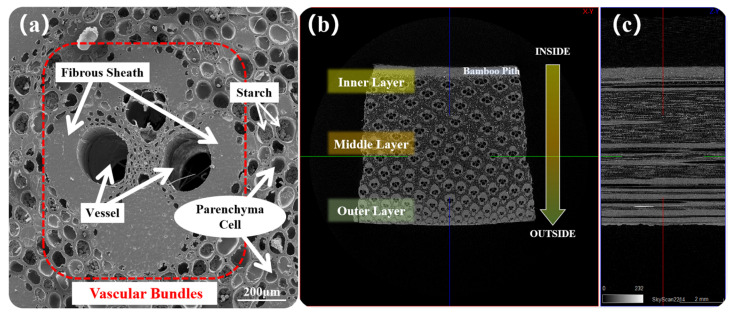
The structure of bamboo sample: (**a**) vascular bundle structure, (**b**) bamboo cross-section structure, and (**c**) bamboo radial-section structure.

A detailed structural analysis of bamboo is presented in Figure 4, where in Figure 4a, the bamboo material is depicted as a composite composed of fibrous sheaths, vessels, and parenchyma cells. Additionally, a significant amount of granular polysaccharide material, typically composed of glucose molecules and serving as a common storage form in plant cells, is observed within the parenchyma cells. Figure 4b illustrates an increasing proportion of vascular bundles from IB to OB. Vascular bundles on the IB side are larger and sparsely distributed, whereas those on the OB side are smaller, densely packed, and exhibit a noticeable gradient structure. Figure 4c offers an additional interpretation of the gradient structure in the longitudinal direction from IB to OB. Vascular bundles, consisting of fibrous sheaths and vessels, show a penetrating distribution. The area of porous regions gradually decreases, with an increasing proportion of fibrous sheath area to the total area. The surface tissue transitions from a loose to a dense configuration, resulting in heightened density and smoothness [7].

**Figure 5:**
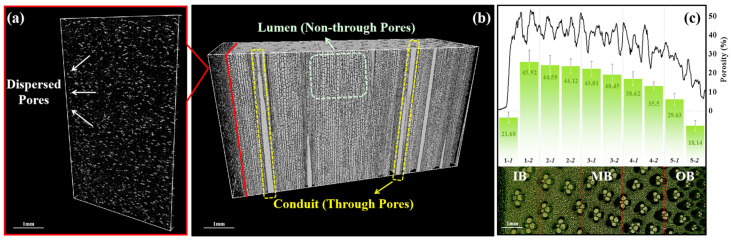
The pore distribution characteristics of bamboo sample: (**a**) chord-section of bamboo pith, (**b**) radial-section of bamboo sample, and (**c**) porosity from IB to OB in cross-section.

The pore distribution characteristics of the bamboo sample are shown in Figure 5; the pore distribution characteristics of the bamboo sample can be categorized into three types in Figure 5b: continuous pores primarily composed of vessels, non-continuous pores mainly composed of parenchyma cells, and intercellular spaces. In Figure 5a, the structure of the bamboo pith is relatively loose, exhibiting evenly distributed pores with a small proportion of approximately 4.2%. Figure 5c analyzes the porosity variation from IB to OB in cross-section, and the data are divided into ten parts based on the gradient structure. The porosity of 1–1 is relatively low at about 21.7%, primarily consisting of parenchyma cells, vessels, and intercellular spaces. Sections 1 and 2 exhibits the highest porosity at 45.9%, mainly composed of parenchyma cells and vessels. As it approaches the OB, the increasing proportion of the fibrous sheath causes the pore proportion to gradually decrease, reaching the lowest value of 18.1% in 5–2 of the OB.

**Figure 6:**
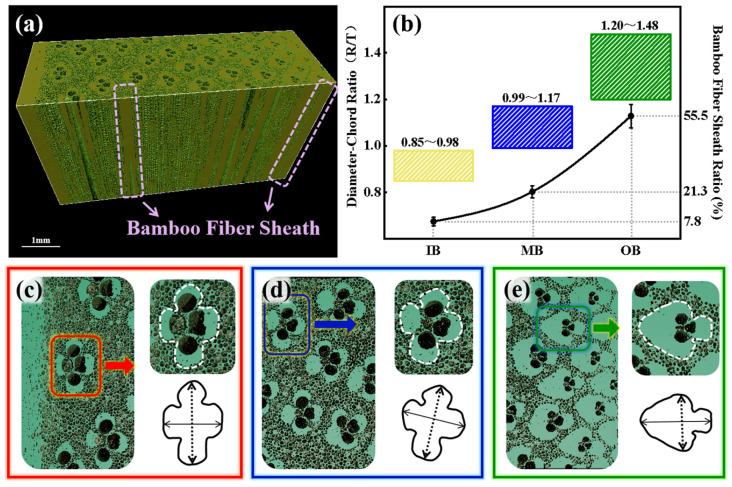
The substance distribution characteristics of the bamboo sample: (**a**) CT image, (**b**) vascular bundle diameter–chord ratio and fiber sheath ratio from IB to OB in cross-section, and (**c**–**e**) morphological evolution of vascular bundles from IB to OB.

The substance distribution characteristics of the bamboo sample are illustrated in Figure 6; the substance distribution characteristics of the bamboo sample show that in the radial-section (Figure 6a), the fibrous sheath exhibits a continuous distribution similar to vessels. In the cross-section, the fibrous sheath exhibits an increasing trend from IB to OB, opposite to the variation in porosity. The morphology of vascular bundles also presents interesting changes in the gradient structure. As shown in Figure 6c, the vascular bundles in IB display basic symmetry in both the diameter and chord directions, containing four similarly sized fibrous sheaths, forming a nearly elliptical “open” structure (with a diameter–chord ratio of 0.85 to 0.98, Figure 6b). As shown in Figure 6d, the vascular bundles in the middle bamboo (MB) are radially symmetrical, with the three inner fibrous sheaths gradually merging, and the inner vessel gradually diminishing. As illustrated in Figure 6e, the vascular bundles in OB are radially symmetrical, with the inner vessel nearly disappearing, containing one large and one small fibrous sheath, forming an arrow-shaped, “semi-open” vascular bundle structure (with a diameter–chord ratio of 1.20 to 1.48).

### 3.2. Chemical Extraction Method Analysis

The summative analysis results of the bamboo are presented in Table 1. OB exhibits the lowest ash content (1.4%), while MB (2.9%) and IB (3.3%) presented a significantly high ash, possibly indicating the presence of more inorganic minerals, such as silica, calcium, or potassium, serving as a potential defense mechanism [34,35], but is less favorable when using bamboo as energy sources for direct combustion, particle manufacturing, and combustion-block production.

The extractive content is highest in the IB at 13.8%, followed by the MB at 11.0%, and is lowest in the OB with 8.7%, showing a gradient descent trend in terms of structure. This may be explained by the presence of more parenchyma cells and less vascular bundles in the IB compared to OB, according to Jia et al. [36]; parenchyma cells have more extractives and hemicelluloses, while vascular bundles have more cellulose. The OB has the highest content of non-polar extractives (0.3%). The polar extractives become the predominant contributor to the total extractive content. From inside to outside, the total content of polar extractives (including ethanol and water extractives) measured 13.4%, 10.9%, and 8.4%, respectively. This phenomenon can be attributed to the structural characteristics of bamboo, where the increase in the relative abundance of thin-walled cells from the IB to the MB (Figure 4a) contributes to the higher content of polar extracts, such as starch granules [15].

The lignin content in bamboo ranged from 19.8% to 24.0%, with OB exhibiting a 4.2% higher lignin content than MB. This transformation primarily signifies the gradual increase in lignification of bamboo from the inner (IB) to outer layer (OB). These values are in the range of values reported by Wen et al. [37] for Klason lignin of moso bamboo stem (25.1%) and pith regions (27.8%). The increase in lignin content enhances the hydrophobicity, structural density, and protective function of bamboo, improving its moisture resistance while maintaining nanoscale mechanical properties [38] and thermal stability [39]. The holocellulose content exhibited comparable variation, ranging from 62.9% to 66.4%. This numerical range is similar to the holocellulose content of Japanese moso bamboo at 65.6% [40], but higher than that of Madu bamboo at 57.3% [35].

### 3.3. Py-GCMS Analysis of Bamboo

**Figure 7:**
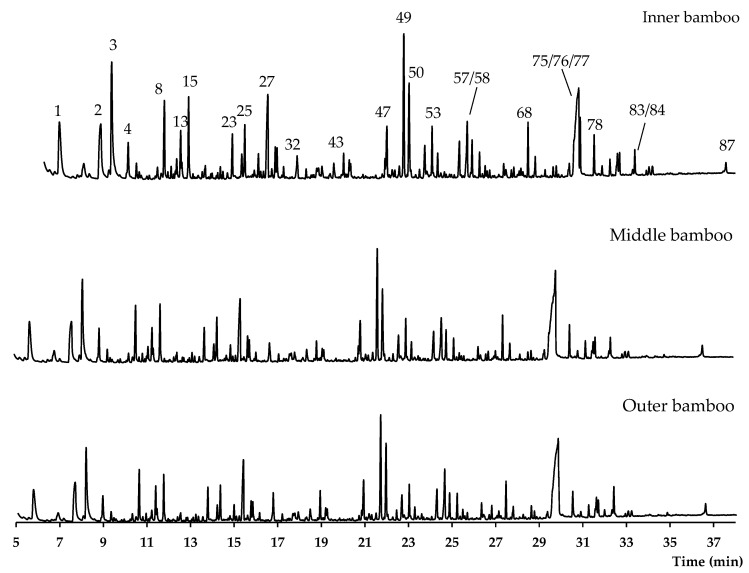
Pyrograms of the bamboo samples. Legend: (1) 2-oxo-propanal; (2) hydroxyacetaldehyde; (3) acetic acid; (4) 1-hydroxy-2-propanone (acetol); (8) 3-hydroxypropanal; (13) CH_3_-CO-CHOH-CHO; (15) furfural and 2-cyclopenten-1-one; (23) 2-hydroxy-2-cyclopenten-1-one; (25) 5-methylfurfural and not-identified sugar; (27) 4-hydroxy-5,6-dihydro-(2*H*)-pyran-2-one; (32) guaiacol; (43) 4-methylguaiacol and not-identified compound; (47) not-identified sugar; (49) 4-vinylphenol; (50) 4-vinylguaiacol; (53) syringol; (57) 1,5-anhydro-B-D-xylofuranose; (58) *trans* isoeugenol; (68) 4-vinylsyringol; (75) 4-propinylsyringol; (76) 1,6-anhydro-β-D-glucopyranose (levoglucosan); (77) *trans* 4-propenylsyringol; (78) syringaldehyde; (83) syringylacetone; (84) *trans* coniferaldehyde; (87) *trans* sinapaldehyde.

**Figure 8:**
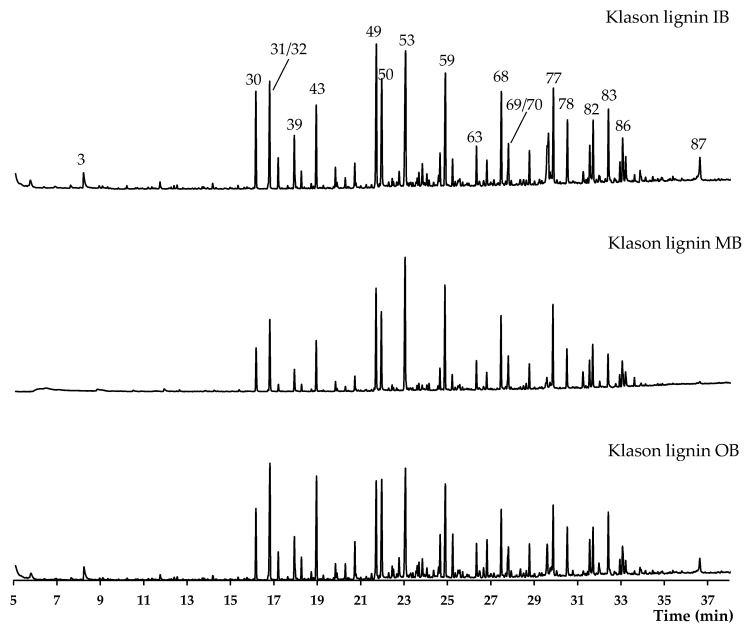
Pyrograms of the Klason lignin from the Klason lignin from bamboo samples: IB—inner; MB—middle; and OB—outer bamboo. Legend: (3) acetic acid; (30) phenol; (31) 4,5-dimethyl-1,3-dioxol-2-one; (32) guaiacol; (39) p- and m-cresol; (43) 4-methylguaiacol and not identified compound; (49) 4-vinylphenol; (50) 4-vinylguaiacol; (53) syringol; (59) 4-methylsyringol; (63) 4-ethylsyringol; (68) 4-vinylsyringol; (69) guaiacylacetone; (70) 4-allylsyringol; (77) trans 4-propenylsyringol; (78) syringaldehyde; (82) acetosyringone; (83) syringylacetone; (86) 3-(3,5-dimethoxy-4-hydroyphenyl)-3-oxopropanal; (87) trans sinapaldehyde.

The bamboo sample (free from extractives) was also characterized by analytical pyrolysis. The pyrograms of the inner, middle, and outer bamboo are presented in Figure 7, and their Klason lignins are presented in Figure 8. The pyrolysis results are presented in Table 2, and in the Supplementary Materials, a complete Table with the identified compounds and their relative percentage is presented.

The inner and outer bamboo presented similar values of carbohydrates content (59.4 and 59.1%), while total lignin reached 25.0 and 27.0%. The middle bamboo sample obtained a similar value of lignin when compared to the outer bamboo (27.2 vs. 27.0%). The samples had a lignin monomeric composition rich in syringyl units (10.5 to 12.7%), followed by guaiacyl units (8.6 to 10.8%) and the *p*-hydroxyphenyl units round 5.8%. Therefore, the H:G:S relation was 1:1.4:1.8 (IB), 1:1.5:2.2 (MB), and 1:1.9:1.9 (OB). The S/G ratio decreased from the inner (1.22) to the outer bamboo (0.99), while the middle presented the highest values (1.46); this may be attributed to a slight increase in S-units in the middle bamboo compared to the IB (42 to 47% of total lignin).

Table 3 illustrates some of the pyrolysis products of bamboo, derivatives from carbohydrates, such as 2-oxo-propanal (peak 1), hydroxyacetaldehyde (peak 2), 4-hydroxy-5,6-dihydro-(2H)-pyran-2-one (peak 27), and levoglucosan (peak 76). The lignin derivatives are composed of H-units (as an example are presented phenol and 4-vinylphenol), G-units (guaiacol and 4-vinylguaiacol), and S-units (syringol and trans 4-propenylsyringol).

As can be seen in Figure 7, no great differences were found between the bamboo samples since all presented the same compounds but with slight differences in relative percentage. In the beginning of the pyrogram, the main compounds are carbohydrates derivatives. The compounds with values higher than 3% were, for example, 2-oxo-propanal (peak 1), hydroxyacetaldehyde (peak 2), acetic acid (peak 3), and 4-hydroxy-5,6-dihydro-(2H)-pyran-2-one (peak 27). After 16 min, the lignin derivatives start to appear, either from H-, G-, or S-skeleton. The lignin derivative compounds with values higher than 3% were 4-vinylphenol (peak 49) and 4-vinylguaiacol (peak 50). At around 30 min, a cellulose derivative, levoglucosan (peak 76) appears overlapped with two lignin derivatives, 4-propinylsyringol (peak 75), and *trans* 4-propenylsyringol (peak 77), as can be seen in Figure 7.

### 3.4. Py-GCMS Analysis of Klason Lignin

In the case of the Klason lignin samples, the pyrograms shown in Figure 8 reveal the presence of almost no carbohydrates; it was only possible to detect acetic acid (peak 3, on average 0.8%) in the inner and outer bamboo samples, as well as some levoglucosan (peak 76, on average 1.3%). The compounds with an average higher than 3% of total chromatographic area were 4-methylsyringol (peak 59, 3.9%), 4-vinylsyringol (peak 68, 4.0%), *trans* 4-propenylsyringol (peak 77, 4.6%), guaiacol (peak 32, 5.3%), 4-vinylguaiacol (peak 50, 5.5%), 4-methylsyringol (peak 59, 5.8%), 4-vinylphenol (peak 49, 6.4%), and syringol (peak 53, 8.8%). Overall, all the lignin samples presented similar amounts of H-units (185—18% of total lignin), while the inner bamboo presented almost twice more S-units compared to G-units (55 *vs*. 26% of total lignin). On the other hand, the outer bamboo presented just slightly more S-units (45 *vs*. 40%). This behavior was more pronounced in milled lignin from bamboo culm (*Phyllostachys pubescens*), where the green region (outer) was rich in G units (54%), followed by S-units (40%), and only 5.9% for H-units, while in the yellow region (inner), the values were 44%, 50%, and 5.2%; an S/G ratio between 0.74 (green) to 1.15 (yellow) was reported using ^13^C and 2D HSQC NMR analysis [41]. These values are lower compared to the values reported for the milled lignin of moso bamboo stem (2.2) and pith (3.7) [37].

Overall, the lignin composition in different bamboo species has been reported as an HGS type of lignin with more G- and S-units compared to H-units, linked together mainly by β-O-4′ aryl ether linkages and with lower β-β′, β-5′ and β-1′ carbon-to-carbon linkages [42]. In particular, for moso bamboo, for the milled lignin, besides the predominance of intermonomeric linkages of the type β-O-4 (45–49 per 100 C 9 units), small amounts of resinols (3.6–7.4), tetrahydrofuran (2.0–2.3), phenylcoumaran (2.8–4.5), spirodienones (1.3–2.3), and α-, β-diaryl ethers were reported (2.8–2.9) [37].

## 4. Conclusions

This study provides a comprehensive analysis of the unique gradient structure, chemical properties, and pyrolysis products of bamboo, offering profound insights for the application and enhancement of bamboo industrial residues. Anatomically, bamboo exhibits a complex gradient structure, from the inner layer (IB) to the outer layer (OB): the IB side has larger, sparsely distributed vascular bundles, resulting in a relatively high porosity of about 45.9%, while the OB side has smaller, densely packed vascular bundles, leading to a decreased porosity of 18.1%. This transition from IB to OB, characterized by an increasing density of vascular bundles and fibrous sheaths, results in enhanced material density and smoothness. Bamboo presented some chemical differences in the radial direction: IB, MB, and OB show a gradient decrease in ashes and polar extractives, but an increase in lignin and holocellulose. Bamboo lignin was SGH type, with a composition rich in S (45–58%) and G-units (26–40%) and low amounts of p-hydroxyphenyl units (15–18%). The lignin composition varied in the three regions studied, where the S/G ratio ranged from 1.22 (IB) to 0.99 (OB). To build on the findings of this research, future studies should focus on developing processing methods that are tailored to the bamboo’s gradient structure and chemical variability. For example, refining pyrolysis techniques to match the specific characteristics of different bamboo regions could enhance the production of high-value bio-products and support more sustainable applications. Additionally, investigating the potential of bamboo-based materials in emerging industries, such as bio-composites or green construction, could further expand its utilization and promote the growth of a circular bioeconomy.

## Figures and Tables

**Table 1:** Chemical composition of bamboo. All values are expressed relative to the dry material (%).

Components (% Dry Material)	Inner Bamboo (IB)	Middle Bamboo (MB)	Outer Bamboo (OB)
**Ash**	**3.3**	**2.9**	**1.4**
**Total extractives**	**13.8**	**11.0**	**8.7**
Dichloromethane	0.2	0.1	0.3
Ethanol	9.1	7.2	4.4
Water	4.5	3.7	3.9
**Total lignin**	**20.1**	**19.8**	**24.0**
Klason lignin	17.8	17.8	22.7
Soluble lignin	2.2	2.0	1.3
**Holocellulose**	**62.9**	**66.4**	**66.0**

**Table 2:** The pyrolysis results of bamboo and Klason lignin samples. All values are expressed as percentage of total chromatographic area.

	Inner Bamboo (IB)	Middle Bamboo (MB)	Outer Bamboo (OB)	Klason IB	Klason MB	Klason OB
Total carbohydrates	59.4	56.4	59.1	5.3	0.8	3.3
Total lignin	25.0	27.2	27.0	75.5	87.8	73.2
Others	0.7	0.7	0.7	0.5	0.5	0.7
Lignin monomeric composition (% of lignin)						
H	24	21	21	18	15	15
G	34	32	40	26	27	40
S	42	47	39	55	58	45
H:G:S relation	1:1.4 1.8	1:1.5:2.2	1:1.9:1.9	1:1.4:3.0	1:1.8:3.9	1:2.7:2.9
S/G ratio	1.22	1.46	0.99	2.10	2.15	1.10
C/L ratio	2.4	2.1	2.2	0.07	0.01	0.04

**Table 3:** Some of the main compounds attained by analytical pyrolysis of bamboo.

Carbohydrates Derivatives		Lignin Derivatives		
2-oxo-propanal (peak 1)	hydroxyacetaldehyde (peak 2)	phenol (peak 30)	guaiacol (peak 32)	syringol (peak 53)
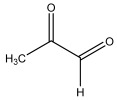	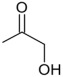	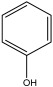	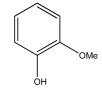	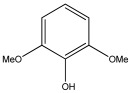
4-hydroxy-5,6-dihydro-(2H)-pyran-2-one (peak 27)	levoglucosan (peak 76)	4-vinylphenol (peak 49)	4-vinylguaiacol (peak 50)	*trans* 4-propenylsyringol (peak 77)
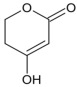	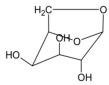	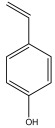	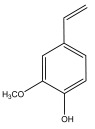	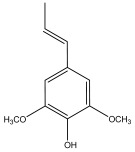

## Data Availability

The original contributions presented in the study are included in the article/Supplementary Materials, further inquiries can be directed to the corresponding authors.

## Supplementary Materials

The following supporting information can be downloaded at https://www.mdpi.com/article/10.3390/ma17205027/s1: Table S1: Identification of carbohydrates and lignin derived pyrolysis compounds (% of total chromatographic area) of the bamboo and Klason lignin.
