# Supplementary material for: Structural and Chemical Analysis of Three Regions of Bamboo (Phyllostachys Edulis)
Source: Materials (Basel). 2024 Oct 14;17(20):5027. doi: 10.3390/ma17205027 (PMC11509333; doi:10.3390/ma17205027)
Supplement: Supplementary file 1 [file materials-17-05027-s001.zip › materials-3160925-supplementary.pdf]

Table S1. Identification and relative quantification of the pyrolysis derived compounds of bamboo samples (values as percentage of chromatographic areas).

Peak nº	RT	Compound		Inner bamboo (IB)	Middle bamboo (MB)	Outer bamboo (OB)	Klason IB	Klason MB	Klason OB
1	5.80	2-oxo-propanal	c	4.4	3.7	3.6	0.7	0.0	0.0
2	7.71	hydroxyacetaldehyde	c	4.7	4.3	4.4	0.2	0.0	0.0
3	8.21	acetic acid	c	6.8	5.8	5.2	1.1	0.0	1.3
4	8.98	1-hydroxy-2-propanone (acetol)	c	1.6	1.6	1.6	0.0	0.0	0.0
5	9.36	HOCH=CHOH	c	0.5	0.5	0.4	0.0	0.0	0.0
6	10.33	CH_2=CH-CO-O-CH_3	c	0.4	0.4	0.4	0.0	0.0	0.0
7	10.51	1,2-ethanediol	c	0.3	0.3	0.3	0.0	0.0	0.0
8	10.65	3-hydroxypropanal	c	2.9	2.6	2.4	0.0	0.0	0.0
9	10.82	CH_2=CH-CO-CHO	c	0.2	0.2	0.2	0.0	0.0	0.0
10	10.96	2(5*H*)-furanone	c	0.4	0.3	0.3	0.0	0.0	0.0
11	11.15	3-Methyl-2(5*H*)-furanone	c	0.2	0.2	0.1	0.0	0.0	0.0
#12	11.23	2(3*H*)-furanone & 3-furaldehyde	c	0.7	0.7	0.6	0.0	0.0	0.0
13	11.41	CH_3-CO-CHOH-CHO	c	1.5	1.3	1.5	0.0	0.0	0.0
14	11.46	succindialdehyde	c	0.6	0.6	0.6	0.0	0.0	0.0
#15	11.78	furfural & 2-cyclopenten-1-one	c	2.6	2.3	2.0	0.3	0.0	0.3
16	12.41	furfuryl alcohol	c	0.2	0.2	0.2	0.0	0.0	0.0
#17	12.54	5-methyl-2(3*H*)-furanone (angelica lactone) & 1-acetoxy-propan-2-one	c	0.5	0.5	0.5	0.0	0.0	0.0
18	12.81	not identified sugar (NI)	c	0.1	0.2	0.1	0.0	0.0	0.0
19	12.87	5-methyl-2(5*H*)-furanone (β-angelica lactone)	c	0.2	0.2	0.2	0.0	0.0	0.0
#20	13.07	furyl methyl-ketone (acethylfuran) & NI sugar	c	0.1	0.1	0.1	0.0	0.0	0.0
21	13.24	not identified sugar	c	0.3	0.3	0.3	0.0	0.0	0.0
22	13.37	4-cyclopentene-1,3-dione	c	0.2	0.2	0.2	0.0	0.0	0.0
23	13.80	2-hydroxy-2-cyclopenten-1-one	c	1.4	1.3	1.4	0.0	0.0	0.0
24	14.23	not identified sugar	c	0.8	0.8	0.7	0.0	0.0	0.0
#25	14.36	5-methylfurfural & NI sugar	c	1.7	1.8	1.5	0.0	0.0	0.0
26	15.01	2(5*H*)-furanone isomer	c	0.7	0.6	0.7	0.0	0.0	0.0

27	15.44	4-hydroxy-5,6-dihydro-(2*H*)-pyran-2-one	c	4.2	4.0	3.7	0.0	0.0	0.0
28	15.78	2H-pyran-2,6(3*H*)-dione	c	0.9	0.9	0.8	0.0	0.0	0.0
29	15.86	not identified sugar	c	0.9	0.8	0.8	0.0	0.0	0.0
30	16.17	phenol	H	0.3	0.3	0.3	3.7	3.3	3.0
31	16.80	4,5-dimethyl-1,3-dioxol-2-one	c	0.5	0.5	0.6	0.0	0.0	0.0
32	16.80	guaiacol	G	0.5	0.5	0.6	4.4	5.1	6.3
33	17.22	*o*-cresol	H	0.1	0.1	0.1	1.1	0.4	1.1
34	17.22	not identified sugar	c	0.1	0.1	0.1	0.0	0.0	0.0
35	17.70	not identified compound	O	0.4	0.3	0.3	0.0	0.0	0.0
36	17.76	methyl-butyraldehyde derivative	c	0.3	0.3	0.3	0.0	0.0	0.0
37	17.79	maltol	c	0.3	0.3	0.3	0.0	0.0	0.0
38	17.95	orcinol	L	0.3	0.3	0.3	0.0	0.0	0.0
#39	17.95	*p*- & *m*-cresol	H	0.3	0.3	0.3	2.0	1.7	2.1
40	18.32	methylguaiacol isomer	G	0.1	0.1	0.1	0.5	0.4	0.8
41	18.31	4-methyl-(5*H*)-furan-2-one	c	0.1	0.1	0.2	0.0	0.0	0.0
#42	18.50	5-hydroxymethyldihydrofuran-2-one & not identified sugar	c	0.7	0.6	0.6	0.0	0.0	0.0
#43	18.95	4-methylguaiacol & NI	G	0.7	0.7	1.1	3.1	3.6	5.0
44	19.21	2,3-dihydroxyhex-1-ene-4-one	c	0.6	0.5	0.5	0.0	0.0	0.0
45	19.27	3,5-dihydroxy-2-methyl-(4*H*)-pyran-4-one	c	0.5	0.5	0.6	0.0	0.0	0.0
46	20.73	4-ethylguaiacol	G	0.1	0.1	0.2	0.9	1.1	1.5
47	20.94	not identified sugar	c	1.9	2.0	1.9	0.0	0.0	0.0
48	21.51	1,4:3,6-dianhydro-α-d-glucopyranose	c	0.5	0.4	0.4	0.0	0.0	0.0
49	21.73	4-vinylphenol	H	4.9	4.7	4.6	6.7	7.7	4.8
50	21.97	4-vinylguaiacol	G	3.3	3.2	3.2	5.3	6.0	5.2
51	22.45	eugenol	G	0.3	0.3	0.4	0.4	0.0	0.0
52	22.70	5-hydrxoymethylfurfural	c	1.2	1.2	1.2	0.0	0.0	0.0
53	23.04	syringol	S	1.3	1.6	1.3	8.0	11.7	6.9
54	23.65	*cis* isoeugenol	G	0.1	0.1	0.1	0.3	0.4	0.5
55	23.71	3-methoxy-5-methylphenol	L	0.1	0.1	0.1	0.5	0.5	0.7
56	24.30	2-hydroxymethyl-5-hydroxy-2,3-dihydro-(4H)-pyran-4-one	c	1.3	1.6	1.6	0.0	0.0	0.0
57	24.66	1,5-anhydro-B-D-xylofuranose	c	1.1	1.2	1.4	0.5	0.4	0.5

58	24.66	*trans* isoeugenol	G	1.1	1.2	1.4	1.1	1.5	1.7
59	24.88	4-methylsyringol	S	1.0	1.2	1.1	5.1	7.7	4.7
60	25.25	vanillin	G	0.6	0.7	0.9	0.9	1.1	1.8
61	25.53	1-(4-hydroxy-3-methoxyphenyl)propyne	G	0.3	0.3	0.4	0.3	0.3	0.3
62	25.74	1-(4-hydroxy-3-methoxyphenyl)propyne	G	0.2	0.2	0.3	0.1	0.3	0.3
63	26.35	4-ethylsyringol	S	0.2	0.3	0.3	1.3	0.9	0.7
64	26.38	homovanillin	G	0.2	0.3	0.3	0.0	0.9	0.7
65	26.70	not identified compound	O	0.2	0.2	0.2	0.0	0.0	0.0
66	26.85	acetoguaiacone	G	0.3	0.3	0.4	0.9	1.2	1.6
67	27.13	4-hydroxybenzaldehyde	H	0.3	0.4	0.3	0.3	0.0	0.0
68	27.47	4-vinylsyringol	S	1.3	1.4	1.2	3.8	5.2	2.9
69	27.80	guaiacylacetone	G	0.1	0.1	0.1	0.4	0.5	0.7
70	27.80	4-allylsyringol	S	0.5	0.5	0.5	1.5	2.1	1.1
71	28.41	propioguaiacone	G	0.0	0.0	0.0	0.4	0.0	0.5
#72	28.66	coniferyl alcohol isomer & guaiacyl vinyl ketone	G	0.3	0.3	0.5	0.2	0.5	0.3
73	28.83	*cis* 4-propenylsyringol	S	0.3	0.3	0.3	0.6	0.9	0.7
74	28.83	α-oxy-propioguaiacone	G	0.0	0.0	0.0	0.6	0.9	0.7
75	29.70	4-propinylsyringol	S	0.9	0.9	0.7	2.1	2.0	1.6
76	29.70	1,6-anhydro-β-D-glucopyranose	c	9.3	10.2	13.7	2.5	0.3	1.1
77	29.92	*trans* 4-propenylsyringol	S	1.4	2.5	0.9	4.1	6.4	3.2
78	30.53	syringaldehyde	S	1.0	1.1	1.1	2.8	2.9	2.4
79	31.26	homosyringaldehyde	S	0.4	0.6	0.5	0.6	1.4	0.0
80	31.61	syringic acid methyl ester	S	0.2	0.2	0.2	1.8	2.0	1.9
81	31.61	not identified sugar	c	0.7	0.7	0.9	0.0	0.0	0.0
82	31.71	acetosyringone	S	0.7	0.8	0.7	2.6	3.2	2.2
83	32.46	syringylacetone	S	0.4	0.4	0.7	3.2	2.6	1.6
84	32.46	*trans* coniferaldehyde	G	0.4	0.4	0.7	0.0	0.0	1.6
85	32.95	propiosyringone	S	0.1	0.1	0.1	0.7	0.8	0.7
86	33.07	3-(3,5-dimethoxy-4-hydroyphenyl)-3-oxopropanal	S	0.3	0.2	0.3	2.2	1.3	0.9
87	36.69	*trans* sinapaldehyde	S	0.4	0.6	0.7	1.5	0.0	1.1
		**total identified**		**85.3**	**84.5**	**87.0**	**81.3**	**89.0**	**77.2**

Total carbohydrates (c)	59.4	56.4	59.1	5.3	0.8	3.3
Total lignin (S+G+H)	25.0	27.2	27.0	75.5	87.8	73.2
Others (O+L)	0.7	0.7	0.7	0.5	0.5	0.7
**Lignin monomeric composition (% of total area)**						
H	**5.9**	**5.8**	**5.6**	**13.8**	**13.1**	**11.1**
G	**8.6**	**8.7**	**10.8**	**19.9**	**23.7**	**29.6**
S	**10.5**	**12.7**	**10.6**	**41.8**	**51.0**	**32.6**
S/G	**1.22**	**1.46**	**0.99**	**2.10**	**2.15**	**1.10**
C/L	**2.4**	**2.1**	**2.2**	**0.07**	**0.01**	**0.04**

RT – retention time (min); # - overlapped compounds; C -carbohydrates derivatives; Lignin derivatives: S – syringyl units; G - guaiacyl units; H – *p*-hydroxyphenyl units; O – other compounds not identified to derive from carbohydrates or lignin.
